# Satellite Hyperspectral Imagery to Support Tick-Borne Infectious Diseases Surveillance

**DOI:** 10.1371/journal.pone.0143736

**Published:** 2015-11-24

**Authors:** Gina Polo, Marcelo Bahia Labruna, Fernando Ferreira

**Affiliations:** 1 Laboratory of Epidemiology and Biostatistics, Department of Preventive Veterinary Medicine and Animal Health, University of São Paulo, São Paulo, Brazil; 2 Laboratory of Parasitic Diseases, Department of Preventive Veterinary Medicine and Animal Health, University of São Paulo, São Paulo, Brazil; University of Texas Medical Branch, UNITED STATES

## Abstract

This study proposed the use of satellite hyperspectral imagery to support tick-borne infectious diseases surveillance based on monitoring the variation in amplifier hosts food sources. To verify this strategy, we used the data of the human rickettsiosis occurrences in southeastern Brazil, region in which the emergence of this disease is associated with the rising capybara population. Spatio-temporal analysis based on Monte Carlo simulations was used to identify risk areas of human rickettsiosis and hyperspectral moderate-resolution imagery was used to identify the increment and expansion of sugarcane crops, main food source of capybaras. In general, a pixel abundance associated with increment of sugarcane crops was detected in risk areas of human rickettsiosis. Thus, the hypothesis that there is a spatio-temporal relationship between the occurrence of human rickettsiosis and the sugarcane crops increment was verified. Therefore, due to the difficulty of monitoring locally the distribution of infectious agents, vectors and animal host’s, satellite hyperspectral imagery can be used as a complementary tool for the surveillance of tick-borne infectious diseases and potentially of other vector-borne diseases.

## Introduction

Active disease surveillance, which involves searching for evidence of disease through routine and monitoring in endemic areas, could help prevent an outbreak, or slow transmission at an earlier stage of an epidemic [1]. Recently, due to the spatial expansion of emerging vector-borne diseases and the difficulty to monitor locally the presence of infectious agents, their vectors and their hosts, epidemiologists are adopting new remote sensing techniques to predict vector habitats based on the identification, characterization and management of environmental variables such as temperature, humidity and land cover type [1, 2]. Based on this strategy, satellite imagery such as those from Advanced Spaceborne Thermal Emission and Reflection Radiometer (ASTER), Landsat, Moderate Resolution Imaging Spectroradiometer (MODIS), and Advanced Very High Resolution Radiometer (AVHRR), have been used to propose preventive strategies for mosquito-borne diseases [3–7]. Ticks are obligatory parasites of vertebrate hosts and depending on their hosts for movement over large distances. Consequently, tick-borne infectious diseases can be spread over geographical areas by the movement of tick hosts carrying either infected ticks or transmitting diseases to susceptible ticks in neighboring locations. In this paper we proposed the use of satellite imagery to support tick-borne infectious diseases surveillance based on the expansion of amplifier host’s food sources.

*Rickettsia rickettsii* is the etiological agent of the most severe form of rickettsiosis, referred to as Brazilian spotted fever (BSF) in Brazil. The disease also occurs in the United States, Mexico, Costa Rica, Panama, Colombia, and Argentina. In South America, the tick *Amblyomma cajennense* sensu lato (s.l.) is the main vector, but other tick species, such as *Rhipicephalus sanguineus* and *Amblyomma aureolatum*, are also involved in more restricted areas [8]. *R*. *rickettsii* is partially pathogenic to ticks so the infection rate drops in the tick population with each tick generation [9]. Furthermore, less than 50% of *A*. *cajennense* s.l. maintain transovarial and transestadial infections and are unable to keep the infection by *R*. *rickettsii* efficiently in successive generations [10]. Thus the participation of amplifier hosts for the maintenance of the bacteria becomes essential to guarantee a constant development of new generations of infected ticks [9, 11]. In South America, it was recently found that the capybara *Hydrochoerus hydrochaeris* acts as the amplifier host of *R*. *rickettsii* infection for the tick *A*. *cajennense* s.l. [12, 13]. Once infected, the capybara keeps the *R*. *rickettsii* in the bloodstream for 7 to 10 days, when the infection of susceptible ticks that feed on it may occur [13], generating new cohorts of infected ticks. In addition capybaras are prolific, producing a mean of six pups per female per year [14], generating a constant introduction of susceptible animals. In North America, where *Dermacentor* spp. ticks are main vectors of *R*. *rickettsii*, several small rodent species such *Microtus pennsylvanicus*, *Microtus pinetorum*, *Peromyscus leucopus* and *Sigmodon hispidus* can also maintained the *R*. *rickettsii* infection between ticks [15].

In Brazil, there may be a causal relationship between the rising capybara population and the re-emergence of the disease, since both capybara populations and the number of BSF occurrences have increased significantly over the last three decades [1, 16]. Between the years 1989 and 2008, there were recorded 737 cases of Brazilian spotted fever with laboratory confirmation, of which 80.2% were in the southeastern region with a lethality of 31.4% [16]. In the state of São Paulo, southeastern Brazil, there were 555 confirmed cases between the years 1985–2012, with a lethality of 40.5% [17]. Additionally, in endemic areas for BSF in the state of São Paulo, population densities of capybaras have reached numbers up to 40 times higher than those recorded in natural environments such as the Amazon and Pantanal [18]. In this region, the constant availability of water resources (S1 Fig), is essential for establishment of capybara populations, since this is a semiaquatic vertebrate species that depends on water source for thermic regulation, reproduction (capybaras mate only in water), and predator protection [19]. On the other hand, the increase of capybara populations in these areas should depend primarily on availability of food sources, as typically known for rodents such as capybaras [19]. Sugar cane is well known as one of the most appreciated food by capybaras, and the economic impact of capybara on damage to sugarcane crops is a reality in the state of São Paulo [20]. In the state of São Paulo, sugar cane is indeed the most common farming [21, 22]. Because sugar cane farming occurs throughout the year, regardless of season, it represents the main food source for capybaras in many areas [18]. Thereby, the increase in the availability of food sources, near watercourses, increases the habitat carrying capacity for capybaras, resulting in population growth of susceptible animals. The carrying capacity represents the maximum number of individuals that the environment can sustain indefinitely given the availability of vital resources (i.e. water, food, habitat) [23]. This in turn could modulate the transmission of diseases carried by these animals.

Among *A*. *cajennense* s.l. populations that are sustained by capybara hosts, it has been proposed that the maintenance of *R*. *rickettsii* depends primarily on a constant introduction of susceptible animals (i.e., newborn capybaras), which will act as amplifier hosts and will guarantee the constant creation of new cohorts of infected ticks [8, 10, 11]. Hence, the approach adopted in this paper is based on the premise that the expansion of cultivated sugarcane areas would translate into an increase of the population density of capybaras (i.e., higher reproduction index), and consequently on the higher exposure of humans to *R*. *rickettsii*-infected ticks (i.e., higher number of BSF cases). In this way, to support the BSF surveillance, we suggest a methodology based on the spatio-temporal monitoring of the increment and expansion of sugarcane crops, main food source for capybaras in the state of São Paulo, southeastern Brazil.

## Materials and Methods

### Study area

To verify if is possible to anticipate the occurrence of tick-borne infectious diseases using the satellite hyperspectral methodology proposed, which is based on monitoring the variation in amplifier hosts food sources, we used the data of the BSF occurrences in the state of São Paulo, southeastern Brazil, region in which the emergence of this disease is associated with the rising capybara population. This study area located at coordinates 19°44′ S to 24°28′ S 44°05′ W to 53°31′ W, covering an area of 248 808 km^2^. With 43,663,669 inhabitants and 625 territorial divisions, São Paulo is the most populous state of Brazil. The topography is characterized largely as a plateau (90%), with altitudes that vary from 300 m to 900 m. The region has a tropical climate, with a hot and humid summer (October to February) and dry winter (June to August).

### Brazilian Spotted Fever occurrence

#### Data collection

The occurrence of Brazilian spotted fever in the state of São Paulo from 2000 to 2012 were obtained from the web site of the São Paulo State Center of Epidemiologic Surveillance [24]. We considered only the BSF cases that occurred in areas of transmission by *A*. *cajennense* s.l., as previously determined [25]. The human population of each São Paulo territorial division was obtained from the website of the Brazilian Institute of Geography and Statistics [26].

#### Retrospective Spatial analysis

A retrospective high rate spatio-temporal statistic based on a discrete Poisson distribution, implemented in a software SaTScan, was used for the identification of spatial clusters and risk areas for BSF occurrence. This method produced a set of clusters, the relative risk in the different clusters, and a corresponding *p*—value for each cluster based on Monte-Carlo simulations.

The statistic uses a circular window of variable radius that moves across the map. For each circle a likelihood ratio statistic is computed based on the number of observed and expected cases within and outside the circle and compared with the likelihood, *L*_0_[27]. We used a likelihood function under the alternative hypothesis assuming Poisson distributed cases proportional to:

$$\begin{array}{c} {\left(\frac{\gamma }{E(\gamma )}\right)}^{\gamma }{\left(\frac{N-\gamma }{N-E(\gamma )}\right)}^{N-\gamma }I\left(\right) \end{array}$$ (1)

where *γ* and *E*(*γ*) represent the observed and expected number of cases in a circle and *N* − *γ* and *N* − *E*(*γ*) the observed and expected number of cases outside the circle. *N* is the total number of cases. The indicator function *I*() is equal to 1 if the observed number of cases within the circle is larger than the expected number of cases given the null hypothesis and 0 otherwise [27]. The circles with the highest likelihood ratio values are identified as potential clusters. An associated *p*-value, based on 999 Monte Carlo simulations, was computed and used to evaluate whether the cases are randomly distributed in space or otherwise. We included only primary clusters, as long as their corresponding *p*-values were less than 0.05.

### Hyperspectral / multitemporal analysis

#### Satellite imagery obtaining

To detect increment and expansion of sugarcane crops we used time series from the Enhanced Vegetation Index (EVI) for the entire state of São Paulo from 2000 to 2012. The EVI imagery were obtained from the Moderate Resolution Imaging Spectroradiometer (MODIS) sensor on board the Terra satellite of the National Aeronautics and Space Administration (NASA) [28]. The gathered images from MODIS, including 176 spectral bands with 250m (red, near-infrared), 500m (mid-infrared) and 1000m (thermal infrared) spatial resolutions. The vegetation spectrum typically absorbs in the red and blue wavelengths, reflects in the green wavelength, strongly reflects in the near infrared wavelength, and displays strong absorption features in wavelengths where atmospheric water is present [29, 30]. These hyperspectral images spans the time period from 2000 to 2012 in 16 day increments. To prepare the images for the analysis, we created a mask to disregard screen brightness values equal to 0 and 255 to rule out specific areas, such as water, in our analyses. All procedures using satellite images were executed using the software ENVI 5.2.

#### Principal Component Analysis

The principal component analysis was used to transform the original hyperspectral image data, highly correlated, into a new set of uncorrelated variables called principal components. By rotating the coordinate system to align with orthogonal dimensions of uncorrelated variance, any location-specific pixel time series *P*_*xt*_ contained in a *N* image time series can be represented as a linear combination of temporal patterns, *F*, and their location-specific components, *C*, as:

$$\begin{array}{c} P_{xt}=\sum_{i=1}^{N}C_{ix}F_{it} \end{array}$$ (2)

where *C*_*ix*_ is the spatial Principal Component (PC), *F*_*it*_ is the corresponding temporal Empirical Orthogonal Function (EOF) and *i* is the dimension [31–35]. The EOF’s are the eigenvectors of the covariance matrix that represent uncorrelated temporal patterns of variability within the data [31]. Thus, the principal components can be determined by computing the eigenvectors and eigenvalues of the covariance matrix.

#### Linear Spectral Unmixing Analysis

The relative abundance of sugarcane crops was depicted in the hyperspectral imagery based on the endmembers spectral characteristics, using linear spectral unmixing. Endmembers are a collection of constituent spectra corresponding to distinct ground substances [36]. Each location-specific (*x*) pixel *P*_*xt*_ in an *N* image time series can be represented as a linear combination of *D*′ temporal endmembers, *E*_*it*_, and a residual component, *ε*, as:

$$\begin{array}{c} P_{xt}=\sum_{i=1}^{D\prime }f_{ix}E_{it}+\varepsilon \end{array}$$ (3)

where the pixel-specific fractions *f*_*ix*_ may represent either the areal fraction of the pixel exhibiting the temporal pattern of the corresponding endmember, or more generally, the Euclidean proximity of that pixel to the corresponding endmember in the temporal feature space. In a temporal unmixing model, each pixel is the linear combination of different temporal endmembers and corresponding fractions [31]. The result is a set of fraction maps representing the spatial distribution of different endmember abundances so, the pixel values indicate the fraction of the pixel that contains the endmember material corresponding to that image.

## Results

**Fig 1:**
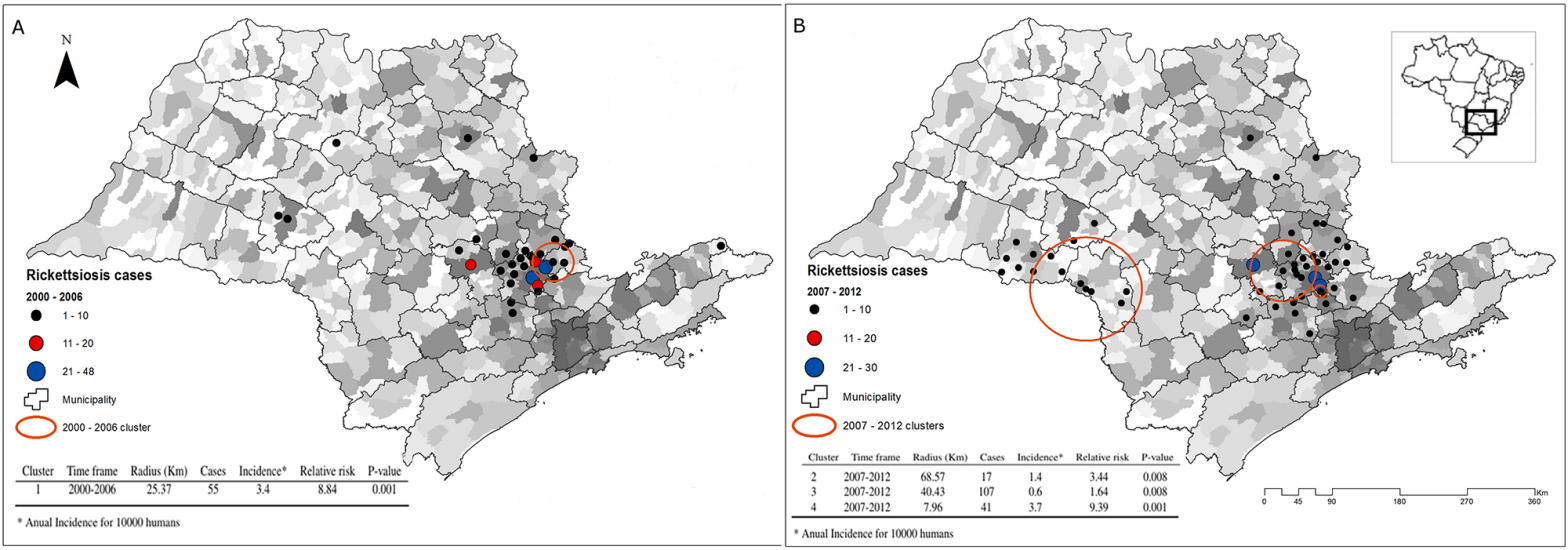
Population density and Rickettsiosis occurrence in areas of transmission by *Amblyomma cajennense* sensu lato in the state of São Paulo. High population density is represented with a higher gray tone and low population density with a lower gray tone. (A) Cases reported and cluster detected from 2000 to 2006. (B) Cases reported and clusters detected from 2007 to 2012.

The distribution of BSF cases in areas of transmission by *A*. *cajennense* s.l. and the São Paulo population density are shown in Fig 1. In São Paulo, from 2000 to 2012 there were reported 386 cases of BSF with an incidence of 0.4 year^−1^ for each 100 000 persons. The retrospective space-time analysis scanning for clusters with high rates using the discrete Poisson model and 999 Monte Carlo simulations detected four spatio-temporal clusters (p<0.05) demarcated in two periods: 2000 to 2006 and 2007 to 2012 as shown in Fig 1. Because of these two temporal clusters found, all following analysis will be presented considering these two periods. Respectively, the number of cases were 178 and 208. The relative risk of the spatial cluster concerning the first period was 8.54 and the relative risk of the three spatial clusters concerning the second period were 1.64, 3.44 and 9.39 as is also shown in Fig 1.

Using principal component analysis, we quantify the spectral dimensionality of the global composite and render the mixing space to select the endmembers. The eigenvalues that derive the amount of variation accounted by each principal component within the feature space, show that nearly 95% of the information within this feature space was found in the first six principal components (66.3% in channel 1, 11.9% in channel 2, 6.3% in channel 3, 4.4% in channel 4, 3.4% in channel 5, 2.4% in channel 6) and for this reason the following analysis considers only the first six principal components. After rendering the mixing space from the six low order principal components we identify three endmembers corresponding to Atlantic vegetation, sugarcane crops and substrates. Substrates includes rock, sediment, soil and non-photosynthetic vegetation and in the case of São Paulo some rivers are also detected as substrates due to the high contamination of water sources. The first four principal components have an annual or biannual frequency change that represents the main vegetation phenology changes over the area. In contrast, the other principal components exhibit poorly expressed annual frequency variability because the corresponding phases stem from noise or substrate, or only represent phenology in small areas. S2 Fig is shown the spectral reflectance information for each endmember selected through the study period. It is shown that the Atlantic vegetation is characterized by an annual cycle, the crop-vegetation by a biannual cycle and the substrate by a poorly annual frequency variability.

**Fig 2:**
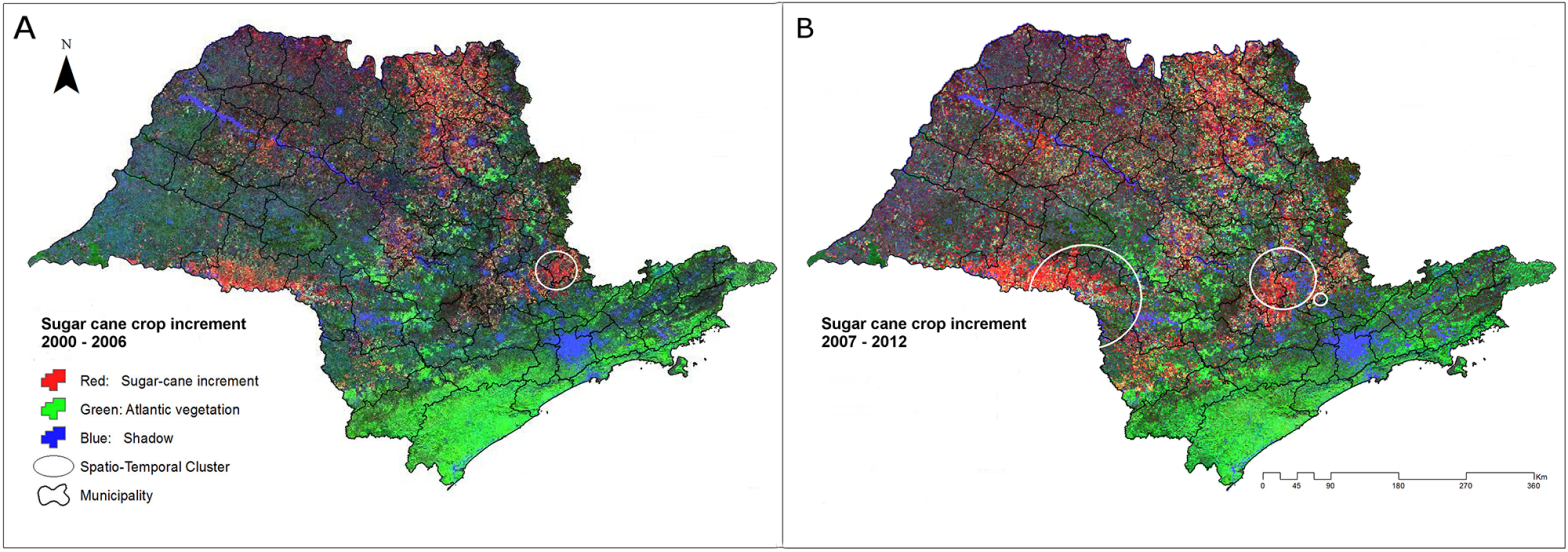
Linear unmixing model result showing the pixel abundance of the endmembers selected from the state of São Paulo. (A) 2000–2006. (B) 2007–2012. Atlantic vegetation is represented in green, sugarcane crops in red and substrates in the blue channel.

Fig 2 represents the pixel abundance of the endmembers selected from the state of São Paulo, using the linear unmixing model. The linear unmixing model yields per pixel endmember fractions which can be interpreted as quantitative estimates of the areal abundance of the specific endmembers (Atlantic vegetation, sugarcane crops and substrates) contributing to the mixed pixel. Thus, in Fig 2 each pixel obtain information of the single mixing fraction that minimize the summed square of the mismatch for all bands using the least square solution. In general, the biannual cycle endmember abundance consistent with sugarcane crops, represented in red, is detected in the cluster areas for rickettsiosis occurrence in both periods. S3 Fig is shown the error map correspondent for each period obtain from the Root Mean Square (RMS) difference between the observed and modeled mixtures. The RMS was used to quantify model misfit and the effects of endmember variability. The RSM mean value for the pixels was 0.318. Black colors indicate that the endmembers chosen for the analysis are well characterized and correspond with the most surface of the state of São Paulo and white colors represent misfit values. Low RMS misfit supports the statistical validity of the linear mixing model but does not guarantee accurate or physically meaningful results. Thus, we verify and confirm the geographical pattern of the sugarcane endmember obtained from joint principal component and linear spectral unmixing analysis with the information of the distribution and expansion of sugarcane crops from previous studies from the state of São Paulo [21, 22] and from the Canasat-Area Project of the Brazilian National Institute for Space Research [37], which mapping the sugarcane distribution of the state of São Paulo once a year using remote sensing imagery acquired by the Landsat, CBERS and Resourcesat-I satellites [37].

**Fig 3:**
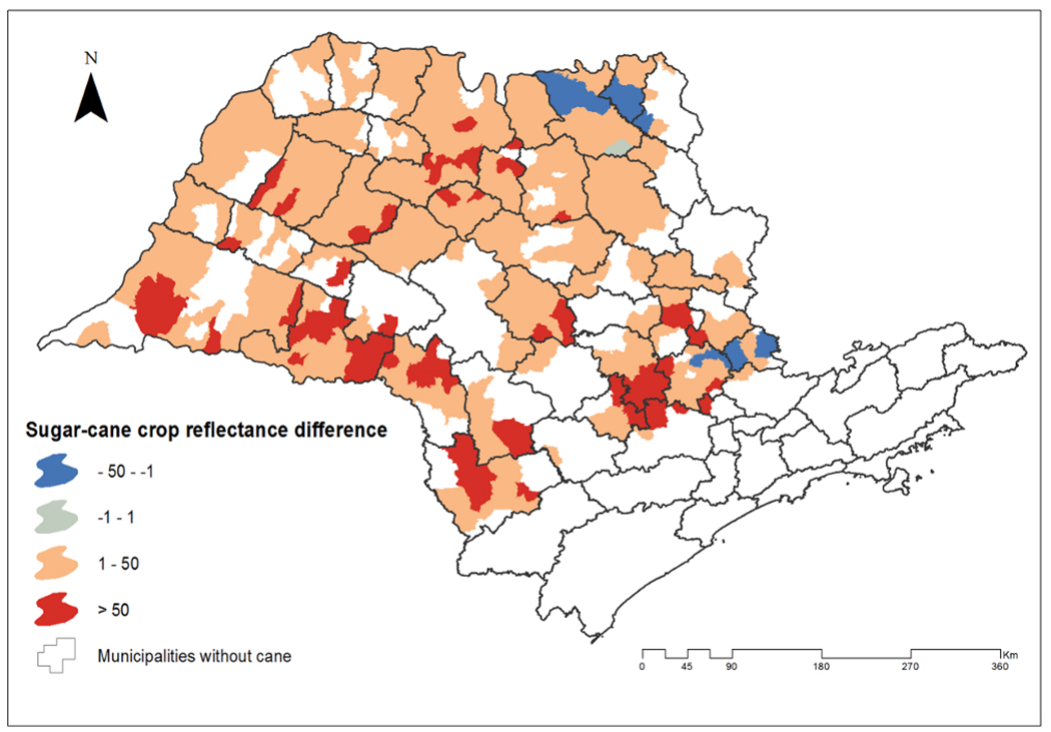
Sugarcane crops pixel abundance difference between the 2007–2012 and 2000–2006 periods. Negative (blue) values indicate that the pixel abundance was higher at 2000–2006 period and positive (red) values indicate that the pixel abundance value was higher at the 2007–2012 period.

To verify if the increment of the sugarcane crops was associated with the presence of BSF, the pixel abundance mean values were compared in the four risk areas for BSF occurrence between both periods. Fig 3 shows the sugarcane crops pixel abundance difference between the 2007–2012 and 2000–2006 periods. To confirm this difference, the sugarcane crops pixel abundance mean values of the clusters was compared between the 2000–2006 and the 2007–2012 periods. It was found that the sugarcane crops pixel abundance mean (124.37) of the three clusters detected from 2007 to 2012 was higher than the pixel abundance mean (88.16) of this zones from 2000 to 2006 (p< 0.05). The sugarcane crops pixel abundance mean (136.81) of the cluster detected from 2000 to 2006 was higher than the sugarcane crops pixel abundance mean (120.13) of this zone from 2007 to 2012, nevertheless this difference was not statistically significant. Consistently, the sugarcane crops pixel abundance mean was 1.4 times higher in the risk zones for BSF occurrence. This indicate that the increment and expansion of sugarcane crops coincide with BSF risk areas. Nevertheless, the reduction of the sugarcane crops not necessarily corresponds with the disappearance of a BSF risk zones.

## Discussion

Spatial epidemiology aims to investigate the spatial distribution of diseases in order to identify risk areas, determine ecological risk factors for disease transmission and strategically guide disease control actions [38, 39]. In our study, four significant high-risk regions for BSF were identified by the spatio-temporal scan statistic approach for two delimited time periods: 2000 to 2006 and 2007 to 2012. The circular scan statistic used in this study has been proved to be useful for many studies [40–43]. Gaudart et al (2005) compared the oblique decision tree model, a complex statistical technique with the Kulldorff’s SaTScan cluster technique, which was used in this study. They produced similar results using both methods in a village in West Africa to identify malaria risk clusters [43]. The circular isotopic technique of the Kulldorff’s SaTScan makes it a useful tool to detect clusters but has limitations when detecting irregular shaped clusters due to its fixed scan window [44, 45].

The suitability of an area for vector-borne disease transmission depends on environmental variables such as temperature, humidity and land cover type. Because these factors can be monitored using remote sensing, it is possible to construct models that predict regions and periods favorable for the presence of the vectors, their hosts and the diseases carried by these. In addition to satellite-derived climate variables, many recent epidemiological studies have made use of the link between vegetation amount and vector populations via the application of normalized difference vegetation index (NDVI) imagery [46]. Due to the essential involvement of the capybaras in the BSF transmission, we used the MODIS enhanced vegetation index (EVI), which offers improvements over the NDVI, to identify sugarcane crops increment and subsequently link this increment with the occurrence of BSF, since we rely on the premisse that higher food availability increases capybara reproduction index, and consequently, higher number of *R*. *rickettsii*-infected ticks [8, 10, 11]. Many remote sensing approaches have been developed to identify cropping practices, such as crop type and cropping intensity, across large spatial and temporal scales [47–50]. Studies have used mainly Landsat data to assess crop type and cropping intensity. However, the accuracy of the Landsat method depends on several different factors such Landsat image availability for the appropriate time period during crop growth cycles [51]. The low temporal resolution of Landsat and its susceptibility to missing values, and the coverage required to assess crop type and cropping intensity is not assured in most agricultural regions, particularly in the tropics where there are often periods of intense cloud covering [52]. High-temporal resolution data from MODIS are of great relevance for modeling the transmission of vector-borne disease since they fill these gaps and allow an assessment of vector and disease distribution and their potential spread.

Studies have been conducted with remote sensing to identify landscapes linked to a higher risk of emerging vector-borne diseases [53, 54]. These studies showed that many factors influence the distribution of ticks and tick-borne diseases, including changes in human activities, biotopes, animal abundance and animal distribution. Therefore, to identify populations, regions or periods at risk for tick-borne diseases, forecasting models must account for many parameters [55]. We demonstrated that areas of BSF occurrence matched significantly with the areas of increment of sugarcane crops, main food source for capybaras, amplifier host for *R*. *rickettsii*. In the state of São Paulo, this single parameter remains constant throughout the year and because of this we do not considered other external factors. In the United States a reduction in the biodiversity due to deforestation and forest fragmentation lead to an increase in the density of white-tailed deer and white-footed mouse and their attendant ticks, leading to the emergence of Lyme disease over the last several decades [4, 6, 56–58].

Our study shows that it is possible to detect high-risk areas for BSF through hyperspectral satellite imagery. We suggest that such analyses will be beneficial by enabling surveillance strategies to be focused on the highest risk regions for BSF. However, several factors need to be considered for these technologies to be routinely adopted for public health management such as, the availability of resources for gathering, processing, and modeling geospatial data, training of personnel on the proper interpretation of results and the continuous availability of remote sensing data in a timely manner [1]. We believe the method could have similar benefits if applied to other vector-borne data collected from other regions and could thus help to improve national surveillance networks for human rickettsiosis.

While our data suggest that the increment in sugarcane crops, observed in Brazil in recent decades had a spatio-temporal relationship with to the occurrence of BSF, our study cannot rule out that other factors that are correlated with the risk of *R rickettsii* infection are also causal for the observed association. Indeed this ecological study does not provide individual-level analysis.

## Conclusion

Our initial hypothesis, that there is a relationship between the sugarcane crops increment and the occurrence of BSF, was objectively reinforced in this paper. In this way, in order to anticipate the occurrence of BSF in the state of São Paulo, we suggest a methodology based on the monitoring of the increment and expansion of sugarcane crops, main food source for the *R*. *rickettsii* amplifier host. Thus, when facing difficulty in monitoring locally the presence of *R*. *rickettsii*, their vectors or their hosts, the remote sensing could help to associate the presence of the disease with environmental changes. The methodology proposed can guide epidemiological surveillance programs in hotspot areas.

## Supporting Information

S1 Fig Hydrography of the State of São Paulo. The uniform distribution of water sources is evident throughout the state.
(TIF)

S2 Fig Spectral reflectance information for the endmembers selected. Spectral reflectance for the Atlantic vegetation (green line), sugarcane crops (red line) and substrates (blue line) endmembers.
(TIFF)

S3 Fig Root mean square (RMS) difference map between the observed and predicted mixtures. White colors represents misfit values and black colors indicate that the endmembers chosen are well characterize.
(TIFF)
