# Supplementary figures and images for: Satellite Hyperspectral Imagery to Support Tick-Borne Infectious Diseases Surveillance
Source: PLoS One. 2015 Nov 24;10(11):e0143736. doi: 10.1371/journal.pone.0143736 (PMC4658071; doi:10.1371/journal.pone.0143736)

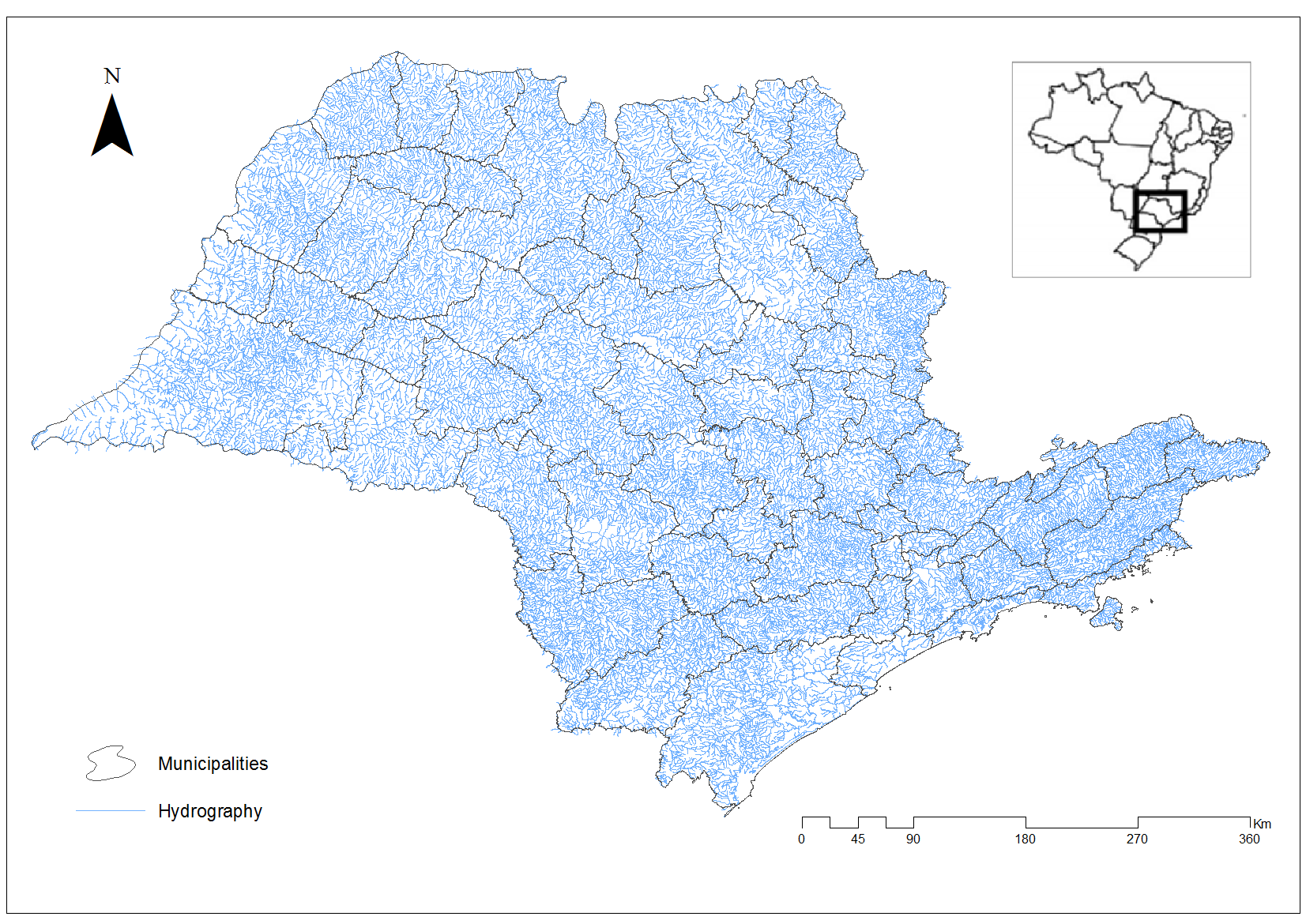

Supplement: S1 Fig — The uniform distribution of water sources is evident throughout the state. (TIF) [file pone.0143736.s001.tif]

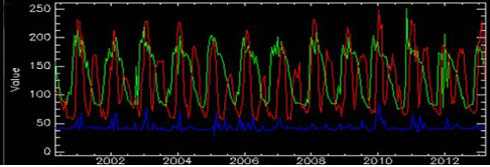

Supplement: S2 Fig — Spectral reflectance for the Atlantic vegetation (green line), sugarcane crops (red line) and substrates (blue line) endmembers. (TIFF) [file pone.0143736.s002.tiff]

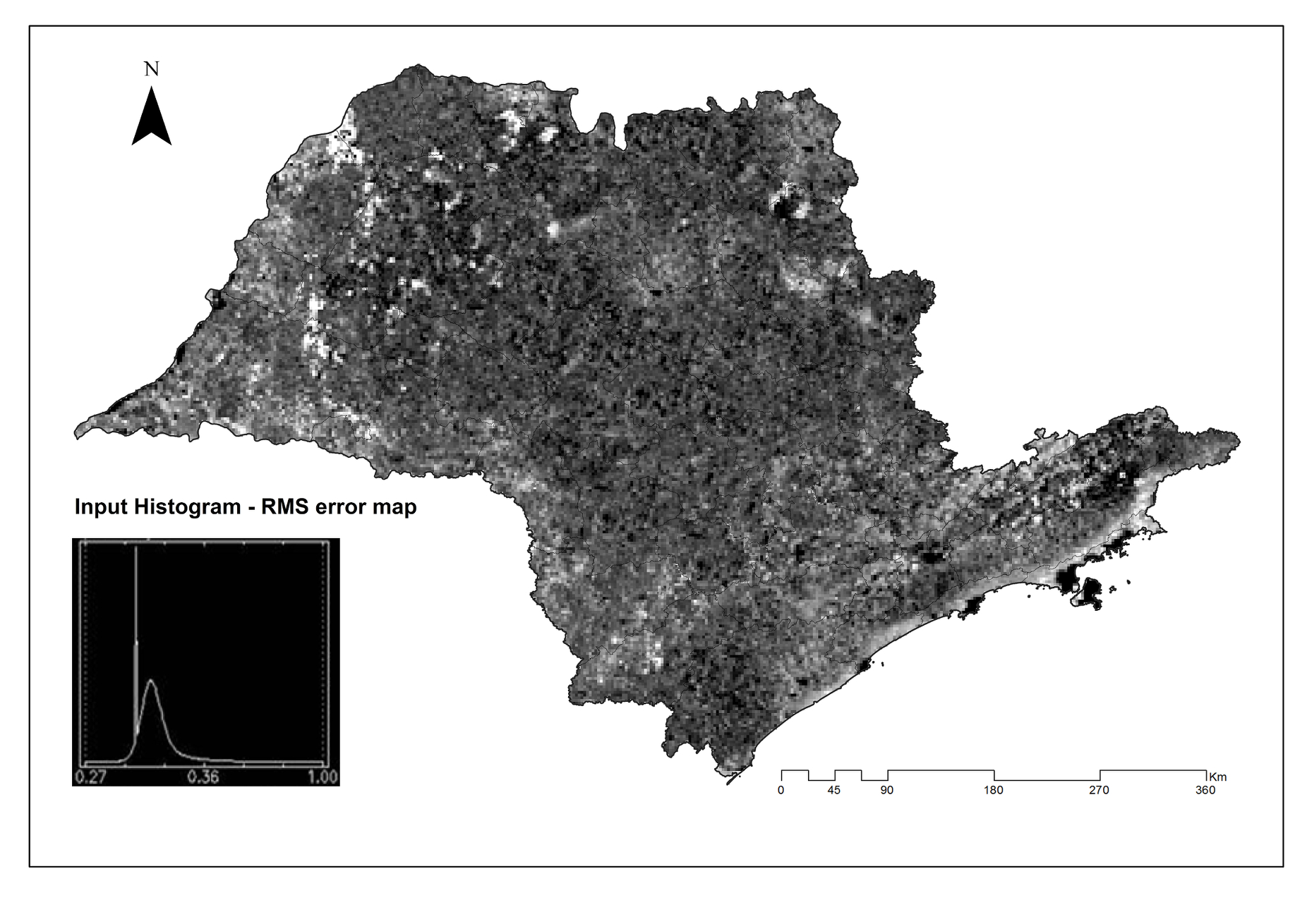

Supplement: S3 Fig — White colors represents misfit values and black colors indicate that the endmembers chosen are well characterize. (TIFF) [file pone.0143736.s003.tiff]
